# Transcriptome Sequencing of and Microarray Development for a *Helicoverpa zea* Cell Line to Investigate In Vitro Insect Cell-Baculovirus Interactions

**DOI:** 10.1371/journal.pone.0036324

**Published:** 2012-05-18

**Authors:** Quan Nguyen, Robin W. Palfreyman, Leslie C. L. Chan, Steven Reid, Lars K. Nielsen

**Affiliations:** Australian Institute for Bioengineering and Nanotechnology, The University of Queensland, St Lucia, Queensland, Australia; University of Kansas Medical Center, United States of America

## Abstract

The Heliothine insect complex contains some of the most destructive pests of agricultural crops worldwide, including the closely related *Helicoverpa zea* and *H. armigera*. Biological control using baculoviruses is practiced at a moderate level worldwide. In order to enable more wide spread use, a better understanding of cell-virus interactions is required. While many baculoviruses have been sequenced, none of the Heliothine insect genomes have been available. In this study, we sequenced, assembled and functionally annotated 29,586 transcripts from cultured *H. zea* cells using Illumina 100 bps and paired-end transcriptome sequencing (RNA-seq). The transcript sequences had high assembly coverage (64.5 times). 23,401 sequences had putative protein functions, and over 13,000 sequences had high similarities to available sequences in other insect species. The sequence database was estimated to cover at least 85% of all H. zea genes. The sequences were used to construct a microarray, which was evaluated on the infection of *H. zea* cells with *H. Armigera single-capsid nucleopolyhedrovirus* (HearNPV). The analysis revealed that up-regulation of apoptosis genes is the main cellular response in the early infection phase (18 hours post infection), while genes linked to four major immunological signalling pathways (Toll, IMD, Jak-STAT and JNK) were down-regulated. Only small changes (generally downwards) were observed for central carbon metabolism. The transcriptome and microarray platform developed in this study represent a greatly expanded resource base for *H. zea* insect- HearNPV interaction studies, in which key cellular pathways such as those for metabolism, immune response, transcription and replication have been identified. This resource will be used to develop better cell culture-based virus production processes, and more generally to investigate the molecular basis of host range and susceptibility, virus infectivity and virulence, and the ecology and evolution of baculoviruses.

## Introduction

The Heliothine insect pest complex, which includes the closely-related *Helicoverpa zea* and *H. armigera* caterpillars, are among the most destructive pests of agricultural crops on a global scale. *H. zea* alone infests at least 30 agricultural crops in North America [1]. The *H. zea* single-capsid nucleopolyhedrovirus (HzSNPV) and the *H. armigera* single-capsid nucleopolyhedrovirus (HearNPV) are effective baculovirus agents often used to control these pests [2] and can be produced in vitro by infecting *H. zea* cells in culture [3]. Baculovirus and insect cell culture technologies are also increasingly being used to produce recombinant proteins [4] and subunit vaccines including virus-like particles [5], and to develop gene delivery vectors including those for cancer therapies [6]–[7]. However, understanding of the interactions between baculoviruses and host cells in culture remains limited, mainly due to a lack of insect genomic sequences. While complete genome sequences for more than 50 baculoviruses are available [8], the genomic information for insect hosts of baculoviruses is poor, with complete genomes only available for the silk worm, *Bombyx mori* [9]. *H. zea* for example has only 191 nucleotide sequences available from the NCBI database as of October 2011. This study applied an effective approach to obtain an almost complete coding sequence database for *H. zea* (via the HzAM1 cell line), so that a comprehensive expression microarray can be built to investigate baculovirus-host interactions.

Insect genome sequencing is challenging due to their large genome sizes (over 430 MB) and other issues such as heterozigosity, transposable elements, and gene duplication [10], [11]. Hence, sequencing is most often performed only for the useful coding regions, rather than for the whole genome. Transcript sequences are conventionally obtained from cDNA libraries, constructed using *E. coli*, from which plasmids are extracted for Sanger sequencing [12], [13], [14]. Recently, the development of deep transcriptome sequencing (RNA-seq) allows massive parallel sequencing of millions of on-chip cDNA libraries, which generates a far higher number of transcript sequences, that can cover a majority of coding genes [15], [16], [17], [18]. The paucity of genomic information for *H. zea* is limiting with respect to quantitative expression analysis using microarrays, since authentic genome sequences are required, which cannot be reliably substituted, even by those of closely-related species [19]. In this study, this problem was circumvented by generating *H. zea* transcript sequences from RNA-seq, which were then used to construct a species-specific genome-scale microarray platform. By combining the best features of both next generation sequencing and microarray technology, this study developed a more affordable approach towards expression analysis for cell production systems lacking genome sequence information. Furthermore, this sequence database can be used more broadly to investigate the molecular basis of *H. zea* insect-pathogen interactions.

This study applied the latest Illumina^®^ sequencing technology to generate millions of raw paired-end and 100 bps long sequences from the *H. zea* transcriptome. Several short-read assemblers, such as Velvet/Oases [20], ABySS [21], [22], SOAP [23], and Trinity [24] have been developed recently for sequence assembly. This study improved assembly by combining the best outputs from two independent assemblers (Oases and ABySS) to generate 29,586 *H. zea* transcript sequences. A number of tools were then used to predict the functions of these sequences (annotation), and a comprehensive hypothetical metabolic network for *H. zea* was constructed to facilitate the analysis of metabolic pathway changes at the genetic level. A large-scale microarray (4×180,000 probes/slide) was then used to experimentally validate the assembled sequences and to select the best probes for a future refined microarray, in which the response of *H. zea* cells to HearNPV infection can be investigated at a more in-depth level. This initial microarray experiment (incorporating probes for both insect and virus genes) was used to analyse RNA extracted from uninfected and HearNPV-infected *H. zea* (HzAM1) cell cultures (harvested at an early infection phase), to provide initial indications of the most significant up-regulated and down-regulated genes and pathways at early virus infection.

## Results

### Sequencing and Assembly of *H. zea* Transcriptome

**Figure 1:**
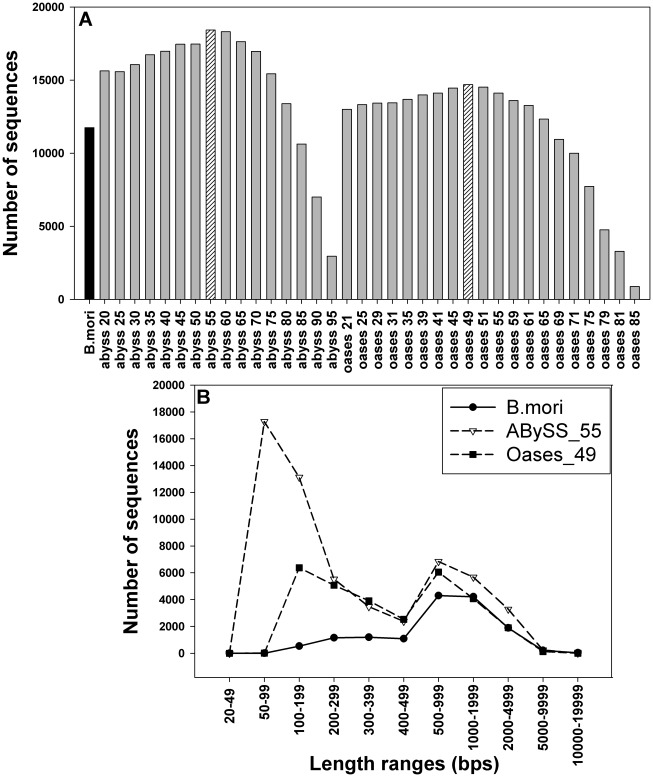
Assessing assembly quality based on lengths and number of contigs. (A) Comparison of total numbers of sequences (longer than 400 bps), generated by ABySS or Oases at different k-mer length parameters. The total number of genes from the model insect, *B. mori*, is shown for comparison. Processed reads were used to run Oases (trimming of 10 last bps and removing of ambiguous quality indicators) and ABySS (no trimming, but removing reads with ambiguous quality indicators) at different k values ranging from 20 to 95 for ABySS and 21 to 85 for Oases. (B) Length distribution of the best Oases and ABySS assembled datasets compared to the *B. mori* database. Numbers of sequences were classified according to length ranges and plotted for comparison. All EST sequences of *B. mori* were downloaded from the silkworm database (http://www.silkdb.org).

The GA II sequencing produced 29,401,474 reads (100 bps per read), with average Sanger quality scores higher than 20, which showed that the base-calling accuracy was above 99% [25]. These high quality reads were assembled independently using Oases and ABySS. The assembly outputs were firstly assessed based on lengths and numbers of sequences, in comparison with the model caterpillar, *Bombyx mori,* which has been fully sequenced (http://www.silkdb.org). In most cases, both ABySS and Oases programs produced more sequences than *B. mori* (**Figure 1**). The numbers of long sequences (larger than 400 bps) were over 13,000 sequences **(** **Figure 1** **A)**. ABySS with k = 55 bps (ABySS_55) generated 57,842 sequences, with an average length of 528.3 bps, while Oases with k = 49 bps (Oases_49) produced 30,006 sequences with an average length of 824.2 bps. Additionally, the sequence size distribution pattern of *H. zea* was compared to that of *B. mori*, showing that both insects have very similar sequence size distributions, in particular having the most sequences at lengths between 500–2,000 bps (**Figure 1** **B**). Since *B. mori* exhibited an insignificant number of ESTs shorter than 200 bps, the assembled *H. zea* sequences that were shorter than this length were also removed. The presence of sequences longer than 10 kb in the *B. mori* dataset suggested that the large sequences obtained from *H. zea* assembly were not abnormal.

**Figure 2:**
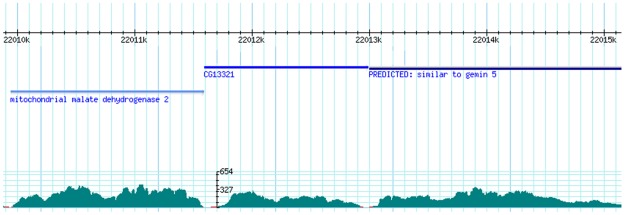
An example demonstrating assembly coverage by mapping reads to a reference scaffold containing assembled sequences. The BWA tools mapped original 100 bp reads to the scaffold of all ABySS_55 sequences with criteria that the matches had > = 95% length and > = 90% identity. In this Figure, the top panel shows reference transcripts. The bottom panel shows coverage as the number of reads per nucleotide position in the reference genes. As shown in this example, many parts of the assembled sequences had coverage higher than 200.

Assembly quality was also assessed by coverage, which is the number of initial raw reads mapped to the final assembled transcripts. The BWA tools [26] mapped 17,805,193 reads (78.7% of clean 100 bps reads) to the assembled sequences, indicating that a large proportion of reads had been assembled into longer transcripts. On average, each ABySS_55 transcript was mapped by 651.7 reads (**Figure 2**). Therefore, the estimated average coverage was 64.5 times, given that the average length of ABySS_55 transcripts was 1,009 bps. This coverage was high, suggesting a reliable assembly output.

*H. zea* genes that have been sequenced previously (available from the NCBI GenBank) were BLAST-compared with the corresponding transcripts assembled in this study, demonstrating that there was over 99% identities in the locally-aligned regions (or High-scoring Segment Pairs - HSPs) between the two datasets (**Table 1**). The HSP regions were longer than 2,000 bps for five out of 15 genes shown in **Table 1**, suggesting that long coding sequences could be assembled accurately. The heat shock protein 70 (HsP70) transcript, for example, had 99.55% similarity, and was 99.1% of the full length of the corresponding *H. zea* gene sequence (2258 bps). However, there were cases that assembled sequences were longer than expected. For instance, Locus_1406 transcript had 2,098 bps (out of total 5200 bps assembled length) alignment to the N-Ethylmaleimide sensitive fusion protein (NSF) (**Table 1**). Further BLAST analysis showed that this transcript matched to a *Manduca Sexta* insect’s intronic mRNA that encodes two proteins, namely NSF protein and Hitcher protein (GenBank accession AF118384), suggesting that the non-aligned parts in assembled sequences may contain useful information. Nevertheless, the whole dataset, 13,381 sequences had long and well-aligned HSP regions, which provided useful information on sequence orientations, accuracy, and functions of assembled transcripts and can be used at high confidence. For example, when compared to 144 top BLASTX matches from the closely related *H. armigera*, 49 protein sequences exhibited 100% identities in the HSP regions (**Table S1**).

**Table 1:** Comparisons of assembled *H. zea* sequences to 15 known *H. zea* sequences.

Protein name	Gene ID	*H. zea* Hits from NCBI database^a^	Identity (%)^b^	Alignment Length (bps)^b^	Assembled length (bps)	Reference Length (bps)^c^
**Lysozyme Precursor**	Locus_26896	FJ535250.1	100	221	223	1048
**Atpase Type 13A1**	Locus_8085	HQ184468.1	99.96	2690	2690	4171
**Ribosomal Protein L13**	Locus_1416	AY846882.1	99.86	365	731	788
**Putative DNA-Mediated Transposase**	103915	DQ788837.1	99.81	1562	1617	2787
**Cytoplasmic Actin A3a1**	Locus_649	AF286060.1	99.74	784	1541	1218
**Casein Kinase I**	Locus_5768	AY220910.1	99.65	1144	1171	1820
**Cytochrome Oxidase Subunit 1**	113509	HQ677772.1	99.57	235	235	700
**Elongation Factor 1-Alpha**	Locus_130	U20136.1	99.56	919	1189	1240
**Heat Shock Protein 70**	Locus_4921	GQ389711.1	99.55	2238	2237	2258
**Acyl-Coa Delta-9 Desaturase**	Locus_518	AF272343.1	99.54	2394	2519	2404
**Dopa Decarboxylase**	Locus_27095	U71429.1	99.39	490	490	690
**Heat Shock Protein 90**	Locus_87	GQ389710.1	99.27	2470	2515	2476
**Heat Shock Protein 70 Cognate**	Locus_572	GQ389712.1	99.25	1593	1624	2082
**N-Ethylmaleimide Sensitive Fusion Protein**	Locus_1406	AY220909.1	99.14	2098	5200	2474
**Apyrase**	Locus_1519	HM569605.1	98.01	1857	3086	2102

a Published *H. zea* sequences available from NCBI GenBank were BLAST to the assembled sequences.

b The completeness and accuracy of assembled sequences were assessed based on sequence identity and the alignment lengths.

c The length of *H. zea* sequences from NCBI GenBank.

BLASTN was applied to assess the reproducibility of de novo assembly and to combine outputs produced by the two independent programs, ABySS and Oases. 24,627 out of 27,402 queries from ABySS _55 (89.9%) had matches in Oases_49, with an average BLAST identity of 99.4%, and an average alignment length of 808.9 (approximately 78.7% of the full length ABySS sequences). Similarly, 22,723 out of 23,682 Oases_49 sequences (96.0%) had matches from ABySS_55. The high proportion of similar sequences between the outputs from these two independent assemblers suggested that de novo assembly had high reproducibility, and hence high reliability. On the other hand, when considering BLAST comparisons to the NCBI databases, Oases_49 sequences had higher similarity and longer alignment length (i.e. better quality), while ABySS produced more hits (i.e. higher quantity). Therefore, to obtain a final dataset that could represent as many *H. zea* genes as possible, 5,904 unique sequences from ABySS_55 were selected from the BLAST to Oases_49 (unique sequences had alignment lengths shorter than 200 bps and identities lower than 95%) and added to the Oases_49 dataset. This combination not only increased the number of sequences by 5%, but also enabled a more comprehensive annotation than those from the original ABySS_55 or Oases_49 datasets alone. As a result, 1,564 putative proteins were added to the Oases_49 annotation.

#### Functional annotation of *H. zea* transcriptome

Full details on putative gene functions, Gene ontology (GO) terms, Enzyme commission (EC) numbers, and KEGG orthologies (KO) for 27,400 selected sequences to be used in the final microarray are shown in Table S1.

**Figure 3:**
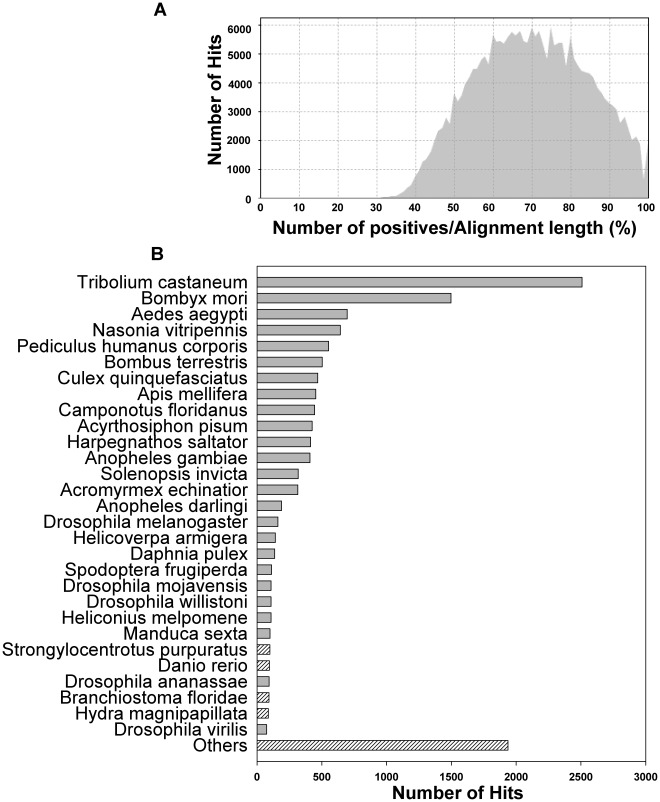
BLASTX similarity distribution and top-hit species distribution. BLASTX was applied for all 29,586 assembled sequences using BLAST2GO (www.blast2go.org/), [27]. (A) Distribution of numbers of sequences at different BLASTX identities. (B) Numbers of top hit sequences from BLASTX were calculated for each species.

From the whole dataset of 29,586 sequences, BLASTX search against the UniProtKB/Swiss-Prot database produced matches for 23,401 sequences (79.1%), among which 13,398 matches exhibited E≤10^−3^. BLASTX was also run on the NCBI non-redundant protein databases using BLAST2GO [27], which produced 13,381 matches with E≤10^−3^. The majority of the best matches with *H. zea* were from insect species, and the similarities mostly ranged between 60 to 80%, which were high given that limited sequences are available from related insects (**Figure 3**). The BLASTX analysis also identified EC numbers for 7,476 sequences. In addition, a BLASTX search against the *B. mori* EST database showed that the *H. zea* transcript set had 14,211 matches (97.2%) from *B. mori* sequences, suggesting a high percentage of *H. zea* genes was obtained.

**Figure 4:**
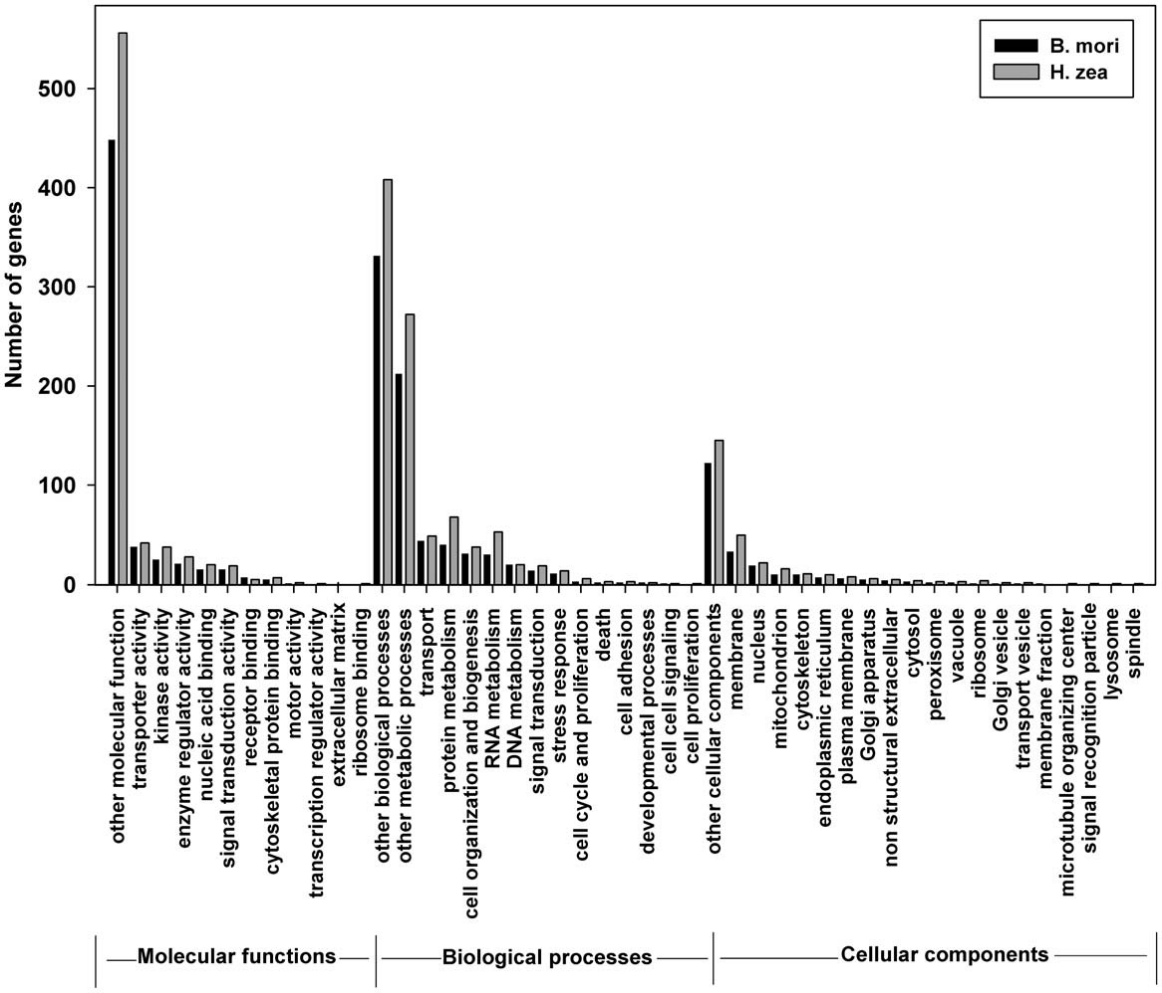
Comprehensiveness of *H. zea* transcriptome as reflected by gene ontology. Gene ontology terms (GOs) for *H. zea* were extracted from InterproScan and were compared to those for *B. mori* downloaded from the silkworm database (http://www.silkdb.org). The GOs were grouped using CateGorizer tool (http://www.animalgenome.org/tools/catego/), [28].

The comprehensiveness of the *H. zea* transcriptome sequences, in terms of functional group coverage, was assessed by classifying transcripts into GO or KO functional groups, and by scrutinizing the putative genes of the immune system and metabolic network. GO terms were assigned using InterProScan on the HMM-Pfam, HMMPanther, and FPrintScan databases. For the *H. zea* sequences, the number of unique transcripts with GO was 5,857 (corresponding to 1,125 unique GO terms), which was comparable to that of *B. mori* (5,971 sequences, corresponding to 961 GO terms). GOs found for all genes were classified into GO_Slim categories (using the CateGorizer tool, http://www.animalgenome.org/tools/catego/, [28]). The distribution of genes to GOs was similar between *H. zea* and *B. mori,* suggesting that the *H. zea* transcript set covers comprehensively all functional categories and that the sequencing and assembly were not biased towards any functional group (**Figure 4**), [28]. In addition, further pathway analysis was performed using the KEGG Automatic Annotation Server (KAAS) (http://www.genome.ad.jp/tools/kaas/), which identified 3,242 *H. zea* sequences with KEGG orthologies (2,806 unique KOs) [29], (**Table S1**). This was also comparable to *B. mori*, which had 3,513 genes with KEGG orthologies (3,071 unique KOs). The comparisons to the fully sequenced genome of *B. mori* in relation to GO and KO terms suggested the comprehensive detection of the *H. zea* transcriptome.

### In-depth Analysis of the *H. zea* Immune System and Metabolic Network

Immune and metabolic pathways are of special interest to both virus production and Heliothine pest management. The *H. zea* transcriptome dataset were investigated further to identify immune and metabolic genes. The dataset had as many immune genes as reported for *B. mori,* which covered all known pathways in the insect innate immune system, including pathogen recognition, modulation, signaling (Toll, Imd, JNK, and Jak/STAT), and effectors (**Table 2** **; Table S2; Table S5**). These genes exhibit high identity with available sequences in the public databases. For example, four out of five *Lepidoptera* caspases (caspases 1, 2, 5, and 6) were identified in this database [30]. Among these four caspases, caspases 1 and 5 had 100% identity, while caspases 2 and 6 had 97% identity, with their homologs in *H.armigera*, respectively. Furthermore, 106 apoptosis-related sequences in *H. zea* were predicted, based on the KEGG apoptosis reference pathway and apoptosis genes of fully sequenced insect species, especially those of the extensively studied *B. mori* [31], (**Table S2**).

**Table 2:** Innate immune genes and apoptosis genes in *H. zea* as compared to B. mori.

Immunepathways	B. mori^a^	H. zea^b^	Up^c^	Down^c^	Unchanged^c^
Recognition	69	48	4	8	36
Modulation	41	26	2	8	16
Toll pathway	27	28	2	12	14
Imd pathway	9	7	1	0	6
JNK pathway	4	7	0	0	7
JAK/STAT pathway	4	5	0	3	2
Apoptosis	73	61	15	8	38

For more details on genes in each pathway, refer to Table S5.

a Numbers of *B. mori* genes were collected from Zhang et al [31], Tanaka et al [57], and from InterProScan annotation (http://www.silkdb.org).

b The numbers listed here are the numbers of different transcript sequences that matched to corresponding proteins from protein BLASTX searches.

c Numbers of *H. zea* genes that were up or down-regulated or unchanged at 18 hours post infection (only up or down regulated genes that had p-adjusted values, which were generated by the Limma linear model with the Benjamini–Hochberg correction method, smaller than 0.05 were counted).

A *H. zea* metabolic network was constructed using Pathway tool to match protein names, EC numbers, and GO terms from BLASTX and InterProScan to metabolic reactions and pathways [32]. The “H.zeaCyc pathway/genome database” linked the genomic repertoire of enzyme genes to the chemical repertoire of metabolic pathways. A web server for accessing this interactive network is available at http://pathway.aibn.uq.edu.au. When the metabolic network of *H. zea* was compared with those from three insects that had fully-sequenced genomes (Pea Aphid, Red flour Beetle and Fruit Fly, http://acypicyc.cycadsys.org/), the *H. zea Cyc* pathway appeared to represent a major part (at least 85%) of the entire insect metabolic network (**Table 3** **)**. Noticeably, the high number of polypeptides for the Pea Aphid as shown in **Table 3** likely contains duplications and redundancy [11], which can be the cause of the higher number of pathways, reactions and enzymes in Aphid than those in the well-annotated Red Flour Beetle and Fruit Fly.

Overall, the database was estimated to cover at least 85% of all *H. zea* genes. This estimation was based on comparisons to *B. mori* in terms of the number of putative proteins (**Figure 1** and **Figure 3**), the coverage and numbers of sequences in different functional groups (**Figure 4**), and number of genes in different immunological pathways (**Table 2** **; Table S5**), and comparisons to *Acyrthosiphon Pisum, Tribolium castaneum and Drosophila melanogaster* in terms of the completeness of the reconstructed metabolic network (**Table 3**).

**Table 3:** Summary of *H. zea* metabolic network^a^ in comparison to other insects that have whole genome sequences (http://acypicyc.cycadsys.org), [58].

Summary metabolic network	*H. zea* (Cotton bornworm)	*Acyrthosiphon pisum* (Pea Aphid)	*Tribolium castaneum* (Red flour beetle)	*Drosophila melanogaster* (Fruit fly)
Pathways	175	207	203	196
Enzymatic Reactions	1,307	1,623	1,568	1,329
Transport Reactions	48	16	12	10
Polypeptides	29,586	34,725	14,462	17,806
Enzymes	2,487	2,967	2,521	3,745
Transporters	173	96	62	144
Compounds	859	1,079	1,025	925

a EC numbers, InterProScan ID, GO terms, and putative protein names of coding sequences were mapped to enzyme groups, metabolic reactions and metabolic pathways to construct a cellular metabolic network.

#### Microarray validation of assembled sequences and selection of probes

A microarray slide that contains 4×180,000 probes was used to experimentally validate assembled sequences, and to select the most suitable probes for a refined microarray platform to be used in future expression analysis of *H. zea*. Six probes were used for each gene target (three probes for each direction) in the microarray. Each probe was tested in four samples, including two for infected and two for uninfected cultures. Among 27,873 sequences that had probes in the array, 27,431 (98.4%) sequences had well above background signal (WABS) in all four samples. Noticeably, 22,773 (81.7%) sequences had WABS for all three probes in a particular direction.

The orientation of *H. zea* transcripts was not obtained from the sequence assembly routine, but was deduced from both the microarray and BLASTX findings. The direction selected for each gene was the one that had the highest cumulative probe signal (from three probes). The probe that produced the highest signal (among the three probes of the selected direction) for each gene target was selected to be included in a refined microarray for expression analysis. Using this approach, the final microarray contains 27,400 probes for *H. zea* sequences and probes for 135 HearNPV virus genes, so that virus-host interactions can be investigated at a genome-scale. The number of probes in this refined *H. zea* microarray is comparable to that of the established *B. mori* microarray platform (which currently consists of 22,987 probes). Sequences of all 27,400 genes and their corresponding probes are presented in the **Table S4**.

#### Differential expression analysis of *H. zea* cell responses at early infection

**Figure 5:**
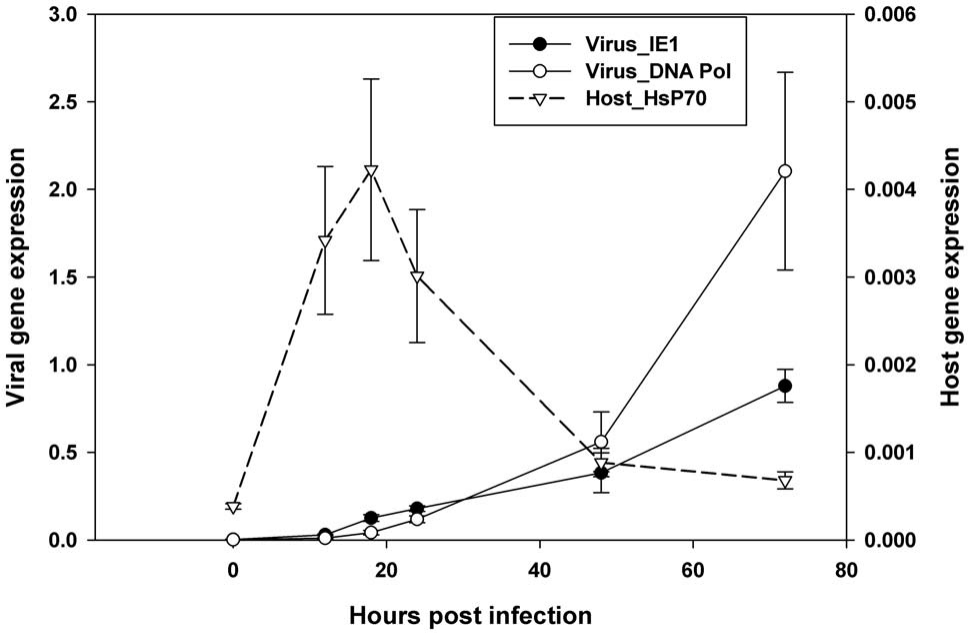
Relative expression of insect and virus genes from 0–72 hours post infection. The expression of a gene at a time point is shown in relative level compared to the reference expression of the 28 S RNA gene at that time [56]. Time course expression level for two early *H.armigera* virus genes, namely DNA polymerase (Pol) and Immediate early gene (IE1) and the host heat shock protein 70 gene (HsP) are shown.

To determine a suitable time for sampling insect cells post infection that exhibited most host responses to infection, time-course expression levels of three early genes (two virus genes and one insect gene) were quantified by RT-PCR at 6 different time points (Figure 5). The viral immediate early 1 gene (IE1) and the DNA polymerase gene express near the beginning after virus entry and are essential for virus replication [8]. On the other hand, in *Spodoptera frugiperda* and *B. mori*, the insect heat shock protein 70 (HsP 70) was consistently found to be a host response gene to viral infection [33], [34]. Therefore, these three genes were selected. The transcription of virus early genes was initiated from 12–24 hours post infection (h.p.i), and increased exponentially after 24 h.p.i. The host response, as reflected by HsP 70 expression, peaked at 18 h.p.i. Thus 18 h.p.i was selected for sample extraction, and microarray signals from the selected probes (27,400 insect genes and 135 virus genes) were utilized to investigate expression changes in cells at an early infection phase.

**Figure 6:**
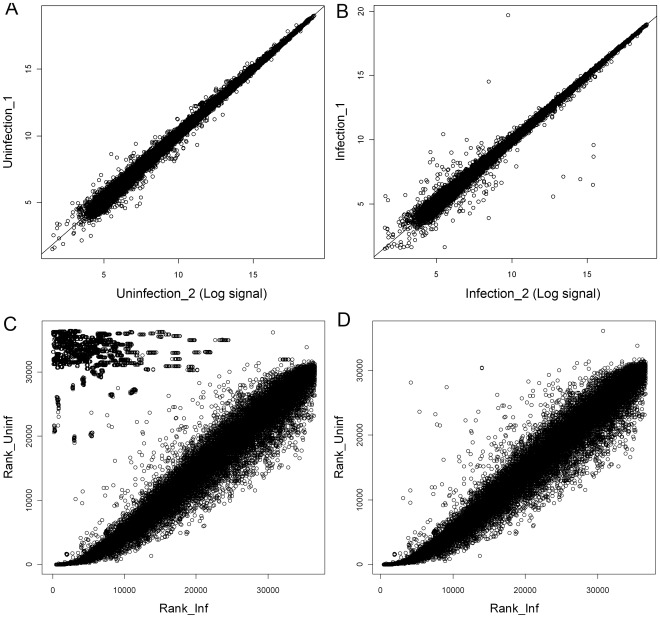
Correlation and distribution of microarray signals. Correlations of microarray signals between two replicated samples for (A) Infected samples 1 and 2 and (B) un-infected samples 1 and 2 were computed using Limma package in R software, [54]. Each spot represents log 2 signal of the same gene in two samples. The high correlation demonstrates the reliability of detection of the microarray platform used. The distribution patterns of gene ranks based on expression levels of all insect-virus genes (C) or insect genes only (D) are shown for assessing the effect of removing virus genes prior to the normalization step.

**Figure 7:**
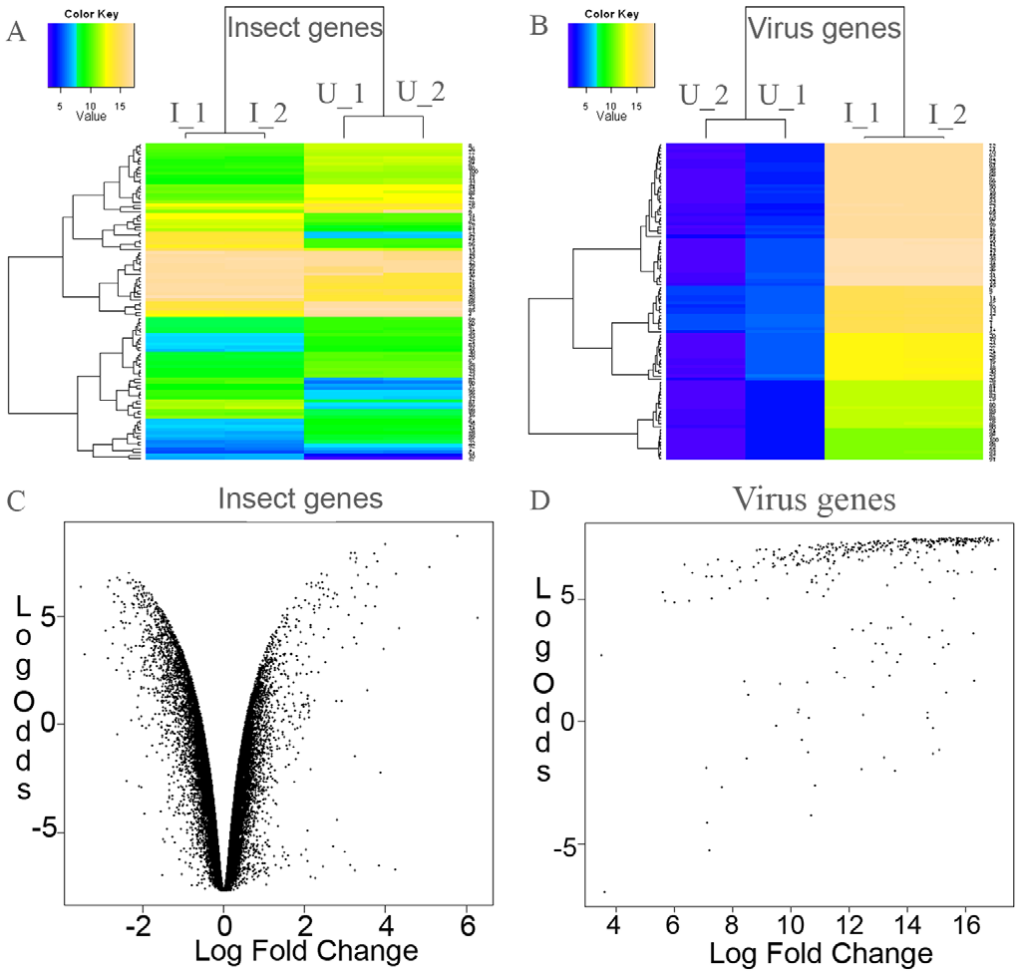
Overview of differentially expressed genes. (A) and (B) show the heatmaps that describe differences in normalized log signal intensity for the first 100 insect genes and 100 virus genes respectively. I1 and I2 are infection 1 and 2, while U1 and U2 are for uninfected samples 1 and 2 respectively. (C) and (D) show overall differential expression profiles for insect genes and virus genes, respectively. The scatter plots show log fold change of expression between two replicates of infected vs. two replicates of uninfected samples (computed by a linear model in LIMMA) and the corresponding Log-odds values. Log-odds is the natural log of the ratio of the probability for the difference being true to the probability of it being not true, i.e. the higher the value the more confidence of difference.

**Figure 8:**
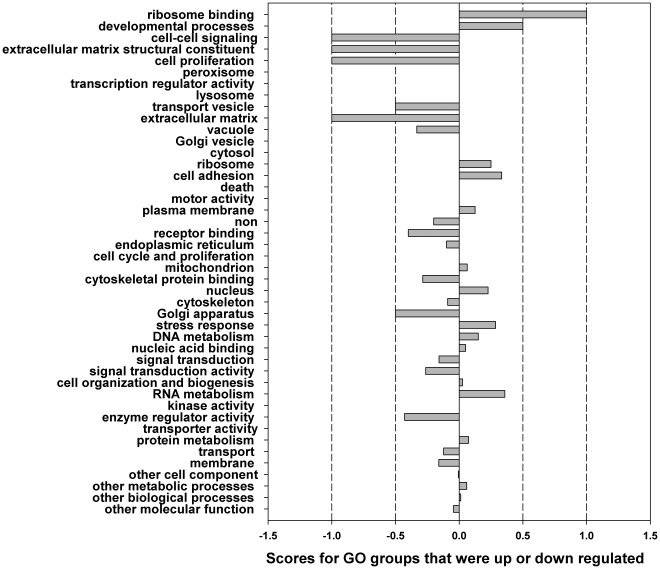
Comparison of numbers of up-regulated genes and down-regulated genes in each GO group, for infected versus non infected samples. Numbers of up-regulated genes and down-regulated genes were both divided by the total number of genes in the GO group that these genes belong to. The ratio for down-regulated genes was then subtracted from the ratio of up-regulated genes to get a score for each GO group. A positive score suggests the group is up-regulated. Only four groups, namely stress responses, RNA metabolism, enzyme regulator activity and Golgi apparatus were statistically confirmed as being significantly different.

Density plots for the microarray signal showed high correlations between duplicates, which were 0.997 for both infected and uninfected replicates (**Figure 6** **A and B**). This demonstrated the high consistency among replicates and the reliability of microarray detection. In order to test the assumption for “Quantile” normalization that the distribution of signals are similar between arrays [35], all genes were ranked according to signal intensity, and the ranks for uninfected and infected cases were plotted against each other (**Figure 6** **C and D)**. Virus genes (top left of the **Figure 6** **C**) had markedly different ranks for uninfected and infected samples, while insect genes had a common distribution pattern between uninfected and infected samples. This suggested that to meet the distribution assumption, the virus genes needed to be analyzed separately from insect genes (**Figure 6** **D**). Linear Models for Microarray Data Analysis (LIMMA) were applied, which showed a high number of up-regulated genes (5,709 or 20.84% had P-adjusted values <0.05 in the linear model), while that of down-regulated genes was 5,313 (19.39%) (**Figure 7** **A and 7 C**). Noticeably, if only the genes with fold-changes higher than 2 times were considered, there were more down-regulated genes (1,062 or 3.88%) than up-regulated genes (442 or 1.61%). On the other hand, virus genes were highly up-regulated (**Figure 7** **B and 7 D**). This large number of regulated genes in *H. zea* was grouped into GO categories. For each category, comparing the number of up-regulated genes to that of down-regulated genes would indicate whether the group was up or down regulated (**Figure 8**). To take into account GO group sizes, numbers of up-regulated genes and down-regulated genes were both divided by the total number of genes in the GO group that these genes belong to. The ratio for down-regulated genes was then subtracted from the ratio of up-regulated genes to get a score for each GO group. A positive score would suggest that the group was possibly up-regulated, while a negative score proposed that the group was likely down-regulated. Figure 8, showed 34 out of 44 such groups. However, when more stringent statistical tests, namely the hypergeometric test and Benjamini–Hochberg correction, were used to confirm these groups, only two groups, namely stress responses (P-adjusted = 0.040) and RNA metabolism (P-adjusted = 0.008), were significantly up-regulated, while the only two down-regulated groups were enzyme regulator activity (P-adjusted = 0.031) and Golgi apparatus (P-adjusted = 0.000). Several high-score groups in **Figure 8** such as ribosome binding (p = 0.429), developmental processes (p = 0.674), cell-cell signaling (p = 0.435), extracellular matrix structural constituent (p = 0.435), and cell proliferation (p = 0.435) could not be confirmed by the statistical tests.

**Figure 9:**
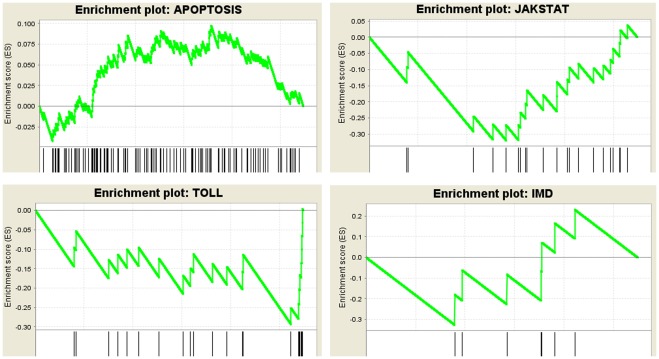
Gene set enrichment analysis (GSEA) of four immune-response pathways in infected *H. zea* cells. A GSEA web-based tool was used (http://www.broadinstitute.org/gsea, version 3.7), [36]. The enrichment score (ES) reflects the degree to which a gene set is overrepresented at the top or bottom of an entire ranked list of genes from the microarray data. A positive ES indicates gene set enrichment at the top of the ranked list (more up-regulated); a negative ES indicates gene set enrichment at the bottom of the ranked list (more down-regulated). Each vertical line in the horizontal axis reflects a gene.

Furthermore, transcripts were selected based on KEGG reference pathways to form five customized gene sets representing immune pathways. These included gene sets for apoptosis, IMD, Jak-STAT, Toll, and JNK pathways. Gene set enrichment analysis (GSEA, http://www.broadinstitute.org/cancer/software/gsea/) was applied to test whether members of each gene set occur randomly or towards the top or the bottom of an entire ranked list of differentially expressed genes (**Figure 9**). Normalized enrichment score (NES, which reflects the degree of overrepresentation of a gene set at the top or bottom and takes into account the size of the gene set), false discovery rate (FDR, which controls the proportion of false positives) and nominal p-value (which estimates statistical significance of NES using gene-set based permutation procedure) were used to identify up/down regulated gene sets [36]. Out of five gene sets, apoptosis was the only one that had a positive NES (NES = 1.61, p = 0.049, FDR = 0.05), while all other four sets had negative NES values smaller than −1.10. Among the down regulated gene sets, Jak-STAT and Toll pathways had a FDR lower than the cut-off value (FDR cut-off  = 0.25 [36]) and significant p-values (p = 0.008, FDR  = 0.057 for Jak-STAT pathway and p = 0.039, FDR = 0.052 for Toll pathway). These results were consistent with the analysis in **Table 2** **(and Table S5)**, which shows that the number of up-regulated genes for apoptosis were double the number of down-regulated genes, while most changes in the Toll pathway and all changes in the Jak-STAT pathway were downward. Together, the data suggested that at an early infection phase one major response of *H. zea* insect cells in culture to virus infection is the up-regulation of the apoptosis pathway. Additionally, expression profiles of genes from the reconstructed metabolic network, especially regarding amino acid or nucleotide degradation and synthesis, energy generation, t-RNA metabolism, and transporters were extensively analyzed (**Table S3**). Overall, most of these genes were not changed or were down-regulated at 18 hours post infection.

## Discussion

### De Novo Assembly of the *H. zea* Transcriptome

The authors successfully assembled reads from one lane sequencing with the recently developed Illumina paired-end and 100 bps technology. There is limited literature on assembling a transcriptome using Illumina short reads (35–75 bps) due to assembly challenges [23], [37]. To date, several *De novo* assembly programs have been released [38]. This study showed that combining outputs from two independent assembly programs such as Oases and ABySS produced better outputs than those using one program only. By using the best outputs from a better assembler such as Oases for the main transcript set, and supplementing these with more unique sequences from other assemblers such as ABySS, a more comprehensive set of transcripts can be derived. A good correlation between the sequences assembled by both programs also enhances confidence in the accuracy of the sequences obtained.

### A Comprehensive *H. zea* Transcriptome

The comprehensiveness of annotation was reflected by the number of protein signatures, GO terms, KO terms, and coverage of the cellular metabolic network and the immune pathways. The enormous sequencing depth of a typical RNA-seq experiment are believed to offer a near-complete snapshot of a transcriptome, including the rare sequences [38]. The results from this study, as well as those from other recent RNA-seq studies, suggest that most mRNAs of the parent organism can be detected from RNA sequencing of cells in culture. In CHO cells [39], more than 98% of 28,914 CHO unigenes were mapped by at least one Illumina read. From cultured human B-cells, Toung et al [40] detected 20,766 genes (over 90%). The *H. zea* transcriptome presented in this study had as many sequences with putative protein signatures and functional categories as those reported for *B. mori*. The *H. zea* Pathway/Genome Database (PGDB) constructed in this study confirmed the presence of most of the key metabolic pathways found in other model insects. This paper is the first to describe the genome-scale metabolic network of a Lepidopteran species. Transcriptomic studies can likely reveal changes in metabolic or transporter genes at the genetic level. Research on the effects of infection on metabolism is emerging, and interesting findings have been reported regarding insect-microbe metabolic interactions [41], [42].

### A Species-specific Microarray for Expression Analysis of *H. zea*

A microarray that is not species-specific does not take into account sequence variation between species, or of strains compared to those from a reference strain, hence while its performance may be acceptable for gene identification, it is inadequate for quantitative expression analysis [19]. This study demonstrated an affordable approach towards expression analysis for an organism without pre-determined genomic sequences, which worked well for *H. zea*. This approach involved RNA-seq to obtain coding sequences, followed by the design of a species-specific microarray for subsequent serial analysis. The microarray platform is relatively low-cost, hence multiple replicates can be analyzed, resulting in well-characterized experimental methodologies, allowing good statistical analysis. The microarray platform can also provide a high level of accuracy, due to recent improvements in the length and number of oligonucleotide probes that can be produced on chips. Furthermore, the use of a large-scale microarray, such as the case in this study, has substantial advantages as more probes can be tested to deduce the direction of sequences, and to minimize the possibility of mis-assembly.

### Response of *H. zea* Cells to HearNPV Infection at an Early Infection Phase

The microarray data suggested that at an early infection phase (18 h.p.i), apoptosis was likely the main response of *H. zea* cells to HearNPV infection, while other immune pathways such as TOLL, IMD, Jak/STAT and JNK were not important defense mechanisms of cultured cells during virus infection. The up-regulation of a number of apoptosis enhancers (including p53 and p53 related genes, cytochrome-c, programmed cell death genes, and caspase genes) as well as the down-regulation of apoptosis inhibitors (including the key IAP gene), provided evidence that cultured *H. zea* cells had triggered apoptosis as a defence against HearNPV virus infection. The decline in IAP levels alone can sufficiently trigger apoptosis [43], [44]. Schultz and Friesen [45], Huang et al. [46] and Vandergaast et al. [44] suggested that Sf9 cells respond to AcMNPV virus infection at an early infection stage upon recognition of virus DNA replication, which causes the depletion of IAP or triggers host cell DNA damage responses, resulting in apoptosis. Likewise, HearNPV-infected *H. zea* cells at an early infection phase, (18 h.p.i., confirmed by RT-PCR), appeared to have induced apoptosis, possibly as a response to signals from HearNPV virus DNA replication.

In conclusion, this study used a state-of-the-art approach to generate a comprehensive database of transcriptome sequences and a microarray platform for *H. zea*, a member of the globally-significant Heliothine insect pest complex of agriculture. This objective was achieved using the latest Illumina sequencing technology, which produced 100 bps and paired-end reads. From the RNA-seq results, an oligonucleotide microarray platform was constructed and validated as a convenient and affordable means of analyzing in vitro insect and baculovirus gene expression simultaneously. Virus-insect interactions are poorly understood. In this study, microarray analysis showed that apoptosis is likely the main response of cultured insect cells to baculovirus infection, at an early infection phase. Hence, virus production may be increased by controlling apoptosis. Furthermore, the transcript sequences and microarray platform generated in this study represent a greatly expanded resource base for *H. zea* insect-pathogen interaction studies in general, in particular to investigate the molecular basis of host range and susceptibility, virus infectivity and virulence, and the ecology and evolution of baculoviruses [47]. This work demonstrates the feasibility to develop a comprehensive transcriptome data base for a complex cell line in a relatively short period of time and at low cost compared to that required to develop a full genome. This coupled with recent advances in microarray technology allows detailed studies of a cell’s response to virus infections to be made. An extension of such work to other cell line/baculovirus systems such as the more commonly used Sf9 and High Five/recombinant AcMNPV systems could rapidly expand our knowledge of what controls the specificity, virion and protein yields of these important in vitro cell culture expression systems.

## Materials and Methods

### RNA Sequencing

Samples containing 5×10^5^ *H. zea* (HzAM1) cells [48], grown in shaker-flask suspension cultures using serum free insect medium (VPM3), were collected for total RNA extraction and DNA removal using the Qiagen RNAeasy extraction and on-column DNAse kit (Qiagen, Hilden, Germany). The cell culture methodology has been described in detail previously [49]. Sequencing was conducted by the Australian Genome Research Facility (Brisbane, QLD, Australia) following the manufacturer’s instructions (Illumina, San Diego, CA). Briefly, the sequencing process included mRNA isolation, cDNA synthesis, adapter ligation, cDNA fragmentation, gel purification (selected a fragment at 214 bps), PCR enrichment of the purified dsDNA library, cluster generation, parallel sequencing by synthesis, and imaging. Clusters were sequenced by two rounds, with 100 cycles per round and one base read per cycle. This generated two of 100 bps reads from each direction for each cDNA fragment.

### Assembly

Two de novo transcriptome assembly programs, Oases 0.1.2 (http://www.ebi.ac.uk/~zerbino/oases/), and ABySS 1.2.0 (http://www.bcgsc.ca/platform/bioinfo/software/abyss), were run using different k-mers ranging from 20 to 95 bps for ABySS and 21 to 85 for Oases [20], [21]. For ABySS, full length reads of 100 bps were used, while for Oases reads with 10 bps trimmed at the end were used. In both cases, reads of vague quality score (those with score B), or of low score (those with scores lower than 20) were removed. Outputs were compared between different k-mer lengths and between two assemblers, based on the numbers and lengths of assembled sequences. The best sets of outputs for Oases and for ABySS were selected, compared and combined to derive a final set that contained all Oases sequences, and extra unique sequences from ABySS that were not present in the Oases output. Short-read data generated in this study was submitted to the NCBI Sequence Read Archive (SRA) database (Accession number SRA048534).

### Annotation

The functions of sequences (i.e. amino acid sequences and protein domains) were predicted via BLASTX2.2.0 search [50] and InterProScan4.6 [51]. Information about protein names, gene ontology (GO) terms, enzyme commission (EC) numbers, BLASTX hits, and InterProScan ID were entered into Pathway-tools13.5 [52], to match sequences with metabolic reactions and pathways, to construct a pathway/genome database (PGDB) for *H. zea*. Pathways of interest were also analysed using information from the Kyoto Encyclopedia of Genes and Genomes (KEGG) database [53] and literature data-mining. All assembled sequences and annotation are presented in the **Table S1**.

### Microarray

A 4×180,000 SurePrint Agilent expression array (Agilent, Santa Clara, CA) was employed so that a high number of probes can be included to test over 27,000 *H. zea* sequences and to eventually select the best probe for each transcript. Six Agilent 60-mer oligonucleotide probes were designed by eArray (Agilent) for each transcript, in which each orientation had three probes randomly distributed across the sequences. Probes that had potential cross-hybridization were removed. The final probe set for *H. zea* sequences included 153,583 probes. In addition, probes for all 135 *H.armigera* single-capsid nucleopolyhedrovirus (HearNPV) genes (three probes per gene) were added to investigate host-virus interactions in culture.

For microarray experiments, total RNA samples (each extracted from 5×10^5^ *H. zea* (HzAM1) cells) were collected from each of two uninfected cultures (seeded at 5×10^5^ cells/mL, harvested at 18 hours post inoculation) and two HearNPV-infected cultures (infected at 5×10^5^ cells/mL with a multiplicity of infection of 17 PFU/cell, harvested at 18 hours post infection, h.p.i.). The cell culture and virus infection methodologies have been described in detail previously [49]. The polyadenylated mRNA of both insect and virus were purified, one-color labeled, hybridized and scanned by the Ramaciotti Centre for Gene Function Analysis (Sydney, NSW,Australia), according to the manufacturer’s instructions (Agilent). Gene differential expression was analyzed using the general linear model in the LIMMA R-package [54]. Signals for virus genes were separated from host genes before normalization. Inter-array variation was normalized using the “Quantile” method [35]. Benjamini-Hochberg method was used for false discovery rate correction, producing P-adjusted values [55]. From the LIMMA output, to identify functional groups that were differentially expressed, genes that had P-adjusted values lower than 0.05 were grouped into different ontology categories using CateGorizer tool [28]. Hypergeometric tests with Benjamini-Hochberg correction were performed for each gene ontology category. More in-depth analysis of immune related genes were carried out using Gene Set Enrichment Analysis 2.0 software [36]. The microarray data have been deposited into the NCBI Gene Expression Omnibus database (GEO accession number: GSE34418) according to MIAME guidelines.

### RT- PCR Expression Analysis

RT-PCR was applied for three genes: the viral IE1 gene (one of the first virus genes to be expressed post infection), the virus DNA polymerase gene (which is expressed at the onset of virus replication), and the insect HsP70 gene (which is used by the insect to respond to virus infection). Total RNA samples, each extracted from 5×10^5^ *H. zea* (HzAM1) cells, were collected from HearNPV-infected cultures (prepared as described previously, with three biological replicates) at 0, 6, 12, 24, 48, and 72 h.p.i. The RNA was extracted using the Qiagen RNAeasy kit (Qiagen). Superscript III, with random hexamers, was applied for cDNA synthesis (Invitrogen, Carlsbad, CA). The epMotion 5075 Robotics System (Eppendorf, Hamburg, Germany) and ABI PRISM® 7900 Sequence Detection System (Applied Biosystems, Foster City, CA) were used for assaying.

## Supporting Information

Table S1 **Annotation of 27,400** ***H. zea*** **transcripts, which were used in the microarray.** For each sequence, a putative protein name, an E-value, the best-hit species, sequence identity and aligment length from protein BLASTX to UniProtKB/Swiss-Prot database or to the NCBI nr databases are listed. Additionally, gene ontology (GO) terms (obtained from InteproScan), KEGG orthologies (obtained from KEGG Automatic Annotation Server) and enzyme commision (EC) numbers (extracted from BLASTX to UniProtKB/Swiss-Prot database) are shown.
(XLSX)

Table S2 **Annotation and microarray analysis of immune genes**. Putative immune related genes were identified based on KEGG pathways and literature data-mining from related insect species that have full genome sequences. For each immune pathway, gene ID, protein names, BLASTX results, and changes in expression level are shown.
(XLS)

Table S3 **Annotation and microarray analysis of metabolic genes.** Putative metabolic related enzymes were predicted by Pathway tools. For each metabolic pathway, gene IDs, protein names, BLASTX results, and changes in expression level are shown.
(XLS)

Table S4 **Transcript sequences, selected probes and log signal intensities in the final microarray platform.** For 27,400 transcripts in *H. zea*, IDs, sequences, selected probes and log signal intensities in all four samples are shown. Full details for 180,880 probes in four samples are available at the NCBI Gene Expression Omnibus database (GEO accession number: GSE34418).
(XLSX)

Table S5 **Innate immune genes and apoptosis genes in** ***H. zea*** **as compared to B. mori.** Numbers of *B. mori* genes were collected from Zhang et al [31], Tanaka et al [57], and from InterProScan annotation. Numbers of *H. zea* genes that were up or down-regulated or unchanged at 18 hours post infection (only up or down regulated genes that had p-adjusted values, which were generated by the Limma linear model with the Benjamini–Hochberg correction method, smaller than 0.05 were counted).
(DOC)
