# Supplementary material for: Transcriptome Sequencing of and Microarray Development for a Helicoverpa zea Cell Line to Investigate In Vitro Insect Cell-Baculovirus Interactions
Source: PLoS One. 2012 May 18;7(5):e36324. doi: 10.1371/journal.pone.0036324 (PMC3356360; doi:10.1371/journal.pone.0036324)
Supplement: Table S5 — Innate immune genes and apoptosis genes in H. zea as compared to B. mori. Numbers of B. mori genes were collected from Zhang et al [31], Tanaka et al [57], and from InterProScan annotation. Numbers of H. zea genes that were up or down-regulated or unchanged at 18 hours post infection (only up or down regulated genes that had p-adjusted values, which were generated by the Limma linear model with the Benjamini–Hochberg correction method, smaller than 0.05 were counted). (DOC) [file pone.0036324.s005.doc]

**Table S5. Innate immune genes and apoptosis genes in H. zea as compared to B. mori.**

| **Recognition** | ***B. mori^a^*** | ***H. zea^b^*** | ***Up^c^*** | ***Down^c^*** | ***Unchanged^c^*** |
| --- | --- | --- | --- | --- | --- |
| C-type lectin | 21 | 12 | 0 | 5 | 7 |
| Draper | 1 | 3 | 0 | 0 | 3 |
| Dscam | 1 | 2 | 0 | 0 | 2 |
| Eater | 0 | 2 | 1 | 1 | 0 |
| Fibrinogen-related protein | 3 | 3 | 0 | 0 | 3 |
| Galectin | 4 | 4 | 0 | 0 | 4 |
| Hemocytin | 1 | 0 | 0 | 0 | 0 |
| Hemolin | 1 | 1 | 0 | 0 | 1 |
| Nimrod A | 1 | 0 | 0 | 0 | 0 |
| Nimrod B | 1 | 2 | 0 | 2 | 0 |
| Nimrod C | 2 | 0 | 0 | 0 | 0 |
| PGRP | 12 | 7 | 1 | 0 | 6 |
| Scavenger receptor A | 18 | 11 | 1 | 0 | 10 |
| TEP | 3 | 1 | 1 | 0 | 0 |
| **Total** | **69** | **48** | **4** | **8** | **36** |
| **Modulation** | ***B. mori*** | ***H. zea*** | ***Up*** | ***Down*** | ***Unchanged*** |
| CLIP serine protease | 15 | 6 | 1 | 2 | 3 |
| Serpin | 26 | 20 | 1 | 6 | 13 |
| **Total** | **41** | **26** | **2** | **8** | **16** |
| **Signaling: Toll pathway, Imd pathway, JNK pathway, and JAK/STAT pathway** | | | | | |
| **(Toll pathway)** | ***B. mori*** | ***H. zea*** | ***Up*** | ***Down*** | ***Unchanged*** |
| Cactus | 1 | 1 | 0 | 0 | 1 |
| Dif/Dorsal | 1 | 1 | 0 | 0 | 1 |
| ECSIT | 1 | 1 | 0 | 0 | 1 |
| MyD88 | 1 | 3 | 0 | 0 | 3 |
| Pelle | 1 | 3 | 1 | 1 | 1 |
| Pellino | 1 | 1 | 0 | 0 | 1 |
| Spätzle | 3 | 3 | 1 | 0 | 2 |
| Toll | 14 | 14 | 0 | 11 | 3 |
| Tollip | 2 | 0 | 0 | 0 | 0 |
| TRAF2 | 1 | 1 | 0 | 0 | 1 |
| Tube | 1 | 0 | 0 | 0 | 0 |
| **Total** | **27** | **28** | **2** | **12** | **14** |
| **(Imd pathway)** | ***B. mori*** | ***H. zea*** | ***Up*** | ***Down*** | ***Unchanged*** |
| Dredd | 1 | 0 | 0 | 0 | 0 |
| FADD | 1 | 1 | 1 | 0 | 0 |
| IKK | 2 | 1 | 0 | 0 | 1 |
| IMD | 1 | 0 | 0 | 0 | 0 |
| Relish | 1 | 2 | 0 | 0 | 2 |
| Tab2 | 1 | 1 | 0 | 0 | 1 |
| TAK1 | 1 | 2 | 0 | 0 | 2 |
| **Total** | **9** | **7** | **1** | **0** | **6** |
| **(JNK pathway)** | ***B. mori*** | ***H. zea*** | ***Up*** | ***Down*** | ***Unchanged*** |
| Fos | 1 | 0 | 0 | 0 | 0 |
| Hem | 1 | 1 | 0 | 0 | 1 |
| JNK | 1 | 4 | 0 | 0 | 4 |
| Jun | 1 | 2 | 0 | 0 | 2 |
| **Total** | **4** | **7** | **0** | **0** | **7** |
| **(JAK/STAT pathway)** | ***B. mori*** | ***H. zea*** | ***Up*** | ***Down*** | ***Unchanged*** |
| Domeless | 1 | 0 | 0 | 0 | 0 |
| Hopscotch | 0 | 1 | 0 | 0 | 1 |
| PIAS | 1 | 1 | 0 | 0 | 1 |
| SOCS | 1 | 1 | 0 | 1 | 0 |
| STAT | 1 | 2 | 0 | 2 | 0 |
| **Total** | **4** | **5** | **0** | **3** | **2** |
| **Apoptosis** | ***B. mori*** | ***H. zea*** | ***Up*** | ***Down*** | ***Unchanged*** |
| Apoptosis-Inducing Factor | 2 | 2 | 2 | 0 | 0 |
| Apoptosis-Linked Protein 2 | 1 | 4 | 0 | 4 | 0 |
| BCL member | 2 | 1 | 0 | 0 | 1 |
| BIRC | 4 | 11 | 1 | 0 | 10 |
| BNIP3 | 0 | 3 | 1 | 1 | 1 |
| Calpain | 1 | 6 | 0 | 0 | 6 |
| Caspases | 4 | 4 | 2 | 1 | 1 |
| CIDE | 3 | 1 | 1 | 0 | 0 |
| FADD | 1 | 1 | 1 | 0 | 0 |
| IAP | 34 | 2 | 0 | 2 | 0 |
| IAP-2 | 2 | 3 | 3 | 0 | 0 |
| IAP-5 | 0 | 2 | 0 | 0 | 2 |
| P53 | 2 | 2 | 1 | 0 | 1 |
| Programmed Cell Death Protein 2 | 1 | 1 | 1 | 0 | 0 |
| Survivin | 16 | 18 | 2 | 0 | 16 |
| **Total** | **73** | **61** | **15** | **8** | **38** |

^a^Numbers of *B. mori* genes were collected from Zhang et al , Tanaka et al , and from InterProScan annotation ([http://www.silkdb.org](http://www.silkdb.org/)).

^b^The numbers listed here are the numbers of different transcript sequences that matched to corresponding proteins from protein BLASTX searches.

^c^Numbers of *H. zea* genes that were up or down-regulated or unchanged at 18 hours post infection (only up or down regulated genes that had p-adjusted values, which were generated by the Limma linear model with the Benjamini–Hochberg correction method, smaller than 0.05 were counted).
